# Enhanced extracellular ammonium release in the plant endophyte *Gluconacetobacter diazotrophicus* through genome editing

**DOI:** 10.1128/spectrum.02478-23

**Published:** 2023-12-01

**Authors:** Benjamin R. Dietz, Neil E. Olszewski, Brett M. Barney

**Affiliations:** 1 Department of Bioproducts and Biosystems Engineering, University of Minnesota, St. Paul, Minnesota, USA; 2 Department of Plant and Microbial Biology, University of Minnesota, St. Paul, Minnesota, USA; 3 Biotechnology Institute, University of Minnesota, St. Paul, Minnesota, USA; State Key Laboratory of Microbial Resources, Institute of Microbiology, Chinese Academy of Sciences, Beijing, China

**Keywords:** *Gluconacetobacter diazotrophicus*, biofertilizer, nitrogen fixation, gene editing

## Abstract

The plant growth-promoting bacterium *Gluconacetobacter diazotrophicus* was originally discovered in association with sugarcane plants as an endophyte. As a member of the small class of organisms defined as diazotrophs, *G. diazotrophicus* is capable of fixing nitrogen from the atmosphere and could serve an important role in minimizing the requirements for nitrogen from industrial-derived fertilizers. In addition to sugarcane, *G. diazotrophicus* is capable of forming endophyte associations with a variety of other important crops. It has been reported that this microbe requires micro-aerobic conditions to effectively fix nitrogen gas from the atmosphere through the enzyme nitrogenase, making it slightly more difficult to study the diazotrophic lifestyle in the laboratory. The ability of the strain to reside within the plant during growth means that any extracellular nitrogen released by this microbe would immediately become available to the plant host. For this reason, it is an ideal target for development as an improved biofertilizer strain. In this work, we constructed strains of *G. diazotrophicus* that result in enhanced ammonium release, as measured by growing with a closely associated algal strain under micro-aerobic conditions, and by further quantifying ammonium concentrations accumulated under micro-aerobic and aerobic growth.

**IMPORTANCE:**

Our results demonstrate increased extracellular ammonium release in the endophyte plant growth-promoting bacterium *Gluconacetobacter diazotrophicus*. Strains were constructed in a manner that leaves no antibiotic markers behind, such that these strains contain no transgenes. Levels of ammonium achieved by cultures of modified *G. diazotrophicus* strains reached concentrations of approximately 18 mM ammonium, while wild-type *G. diazotrophicus* remained much lower (below 50 µM). These findings demonstrate a strong potential for further improving the biofertilizer potential of this important microbe.

## INTRODUCTION

Nitrogen is a primary nutrient for biomass production and is important to crop yields (1, 2). The Haber–Bosch process was initially devised to overcome the limitations in the availability of ammonia, and one result of this process was the ushering in of the green revolution, resulting in a rapid increase in the productivity of many agricultural crops (3). The downside of this process is related to the extensive energy that is required to drive it (4). The Haber–Bosch process uses high temperatures and pressures to convert nitrogen gas, and typically hydrogen derived from methane, into ammonia. Nitrogen is commonly applied to agricultural fields as a conventional synthetic fertilizer in the forms of ammonia, urea, or nitrate (5 – 7).

Biological nitrogen fixation (BNF) is accomplished through the enzyme nitrogenase which is found in a subclass of microbes referred to as diazotrophs (8, 9). Specific model diazotrophic microbes have been recognized for many decades (10 – 12). Enhancing the production of extracellular nitrogen production in diazotrophs is viewed as one potential solution to lowering our dependence on industrial fertilizers and developing more sustainable options to feed a growing population (13 – 15).

Prior efforts to increase extracellular nitrogen production in diazotrophs have focused on a limited number of model microbial strains (13, 16 – 22). Our laboratory has concentrated primarily on *Azotobacter vinelandii* as a potential biofertilizer strain (13, 23 – 27), as it has a nice repertoire of genetic tools for manipulation (28, 29), and is also able to fix nitrogen while growing aerobically. This aerobic growth differentiates *A. vinelandii* from many other strains that require anaerobic or micro-aerobic conditions for BNF. The ability to fix nitrogen while growing aerobically enables the growth of *A. vinelandii* in co-culture with various algal strains (13, 21, 27). Algae have been touted as a potential next-generation bioenergy crop, but the growth of algae (aside from a small subset of blue-green algae known as cyanobacteria), would still require significant inputs of nitrogen to support biomass production (30, 31). One drawback to *A. vinelandii* is that it is a free-living soil diazotroph that does not generally form tight associations with higher land plants (12, 32). As an alternative, we have selected to translate our prior successes with *A. vinelandii* biofertilizer development to *Gluconacetobacter diazotrophicus*, a microbe that was originally discovered in sugarcane crops (33). In sugarcane, *G. diazotrophicus* is found as an endophyte living within the tissues of the plant. *G. diazotrophicus* has since been shown to associate with a number of additional crops including Arabidopsis, wheat, sorghum, and coffee, either naturally, or following experimental crop inoculation (34 – 39). This endophyte lifestyle makes *G. diazotrophicus* a promising target for the potential enhancement of biofertilizer potential by applying approaches to increase extracellular nitrogen production.

**Fig 1:**
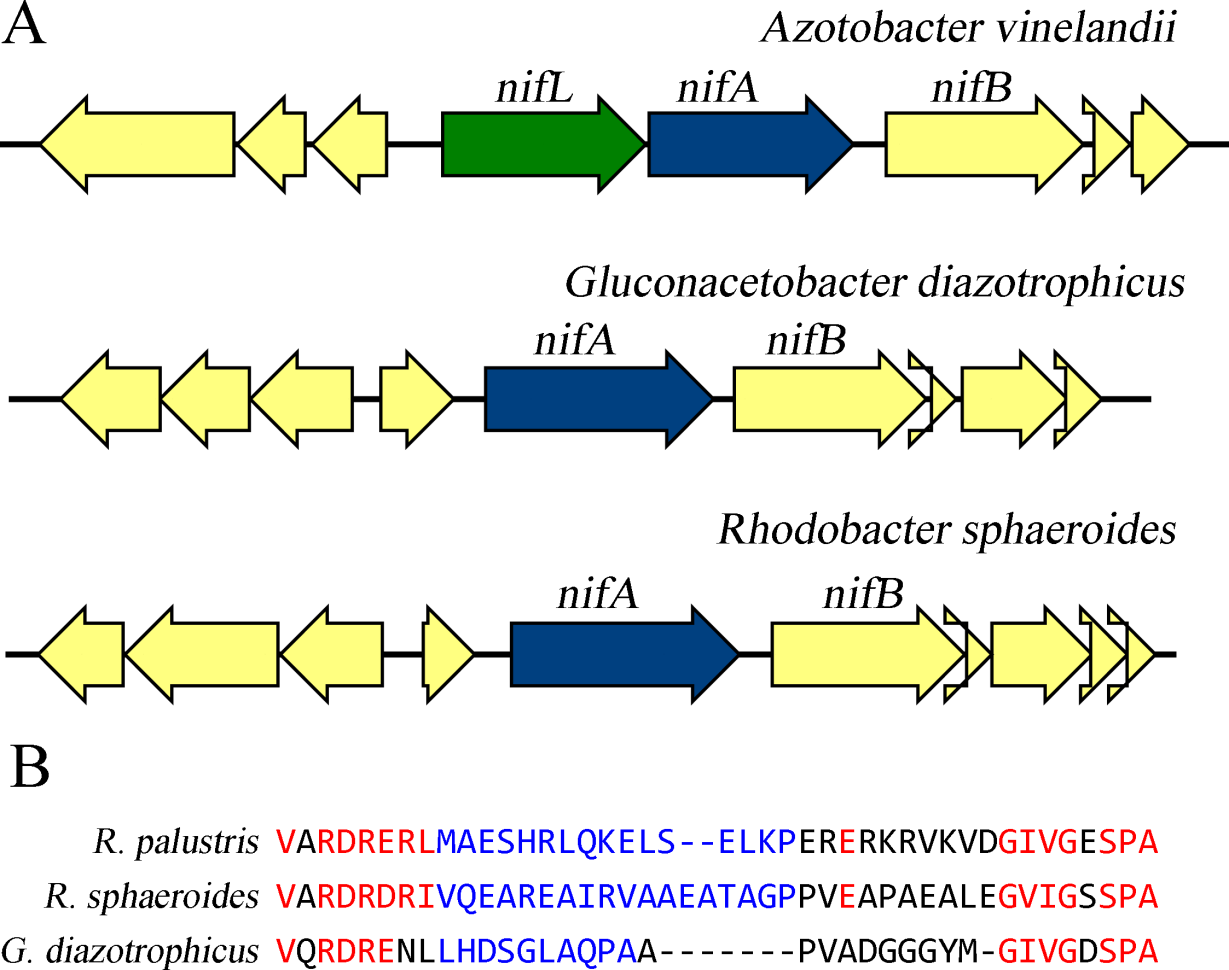
Genome architecture of nitrogen regulator genes *nifA* and *nifL*. Shown above are segments of the genomes for *A. vinelandii*, *G. diazotrophicus,* and *R. sphaeroides* (recently renamed *Cereibacter sphaeroides*). *A. vinelandii* contains genes for both elements of the nitrogen regulator system, NifL, and NifA. *G. diazotrophicus* does not contain a *nifL* gene, similar to *R. sphaeroides* (**A**). The Q-Linker region near the center of the NifA primary amino acid sequences from *R. palustris*, *R. sphaeroides,* and *G. diazotrophicus* are shown in blue, while highly conserved residues are shown in red (**B**). The Q-Linker regions of *R. palustris* and *R. sphaeroides* were targeted previously to enhance hydrogen production through nitrogenase (45, 47). The predicted Q-Linker segment targeted as part of this work with *G. diazotrophicus* is also shown.

Strategies to increase extracellular ammonium production in *A. vinelandii* have included efforts to delete the ammonium transporter AmtB, and derepression of the master regulator NifA through disruption of *nifL* that encodes the NifA partner protein NifL which modulates NifA activity (13, 40, 41). Ammonium that leaks across the membrane as a result of limited diffusion is generally recaptured by the ammonium transporter AmtB (13, 18, 26, 42, 43). Additional approaches have targeted ammonium assimilation through substitutions to *glnA* that decrease glutamine synthetase activity or alter glutamine synthetase activity through post-translational modification (41, 44). In contrast to *A. vinelandii*, the *nifA* regulatory system of *G. diazotrophicus* lacks a *nifL* component, more similar to the regulatory systems that are found in *Rhodobacter sphaeroides*, *Rhodopseudomonas palustris*, and *Azorhizobium caulinodans* [Fig. 1 and (45 – 47)]. Additionally, *G. diazotrophicus* contains two separate homologs of the *amtB* gene with high conservation within the primary protein sequence (92% identical for the core protein sequence), while *A. vinelandii* only contains one copy of *amtB* (48, 49). These multiple copies of *amtB* in *G. diazotrophicus* (48, 50) likely minimize the potential loss due to unintentional mutations arising in either single copy of the gene and may work together to ensure minimal ammonium losses. The differences in genome architecture require an alternative approach to increase extracellular ammonium production in *G. diazotrophicus* versus what has previously been successful in *A. vinelandii*.

In this report, we detail efforts to increase extracellular nitrogen production through enhancing ammonium release and relieving NifA repression of BNF in *G. diazotrophicus*. Both processes were modified and then combined to generate a progression of strains that had increased extracellular nitrogen release. The contrast between the findings of this work and what has been done in previous studies and with previous strains is presented.

## MATERIALS AND METHODS

### Bacterial strains and growth conditions

*G. diazotrophicus* PA1 5 (ATCC 49037) was obtained from Cedric Owens and grown aerobically at 30°C on GADN medium (all per liter; 2 g glucose, 2 g yeast extract, 1.5 g tryptone, 0.5 g MgSO_4_⋅7H_2_O, 1.5 g sodium glutamate, 200 mg ammonium sulfate, and 200 mg asparagine, adjusted to pH 6.2 with HCl (51)). For diazotrophic growth plate experiments, a modified Burk’s (B) medium was utilized that contained 50 mM sodium citrate as buffer, 20 g sucrose, 200 mg MgSO_4_⋅7H_2_O, 90 mg CaCl_2_⋅2H_2_O, 0.25 mg Na_2_MoO_4_⋅2H_2_O, 5 mg FeSO_4_⋅7H_2_O and 2 mL of a phosphate buffer composed of 20 g KH_2_PO_4_ and 80 g K_2_HPO_4_ in 1 L dH_2_O; adjusted to pH 6.2 with NaOH or HCl. GADN media used to isolate *G. diazotrophicus* following plasmid manipulations was supplemented with 100 mg L^−1^ tetracycline and/or 200 mg L^−1^ kanamycin. When grown together with algae, this medium was further supplemented with 1 mL per liter of micronutrient solution (13, 52) and substituted with plant growth grade agar.

### *G. diazotrophicus* strain construction

**Fig 2:**
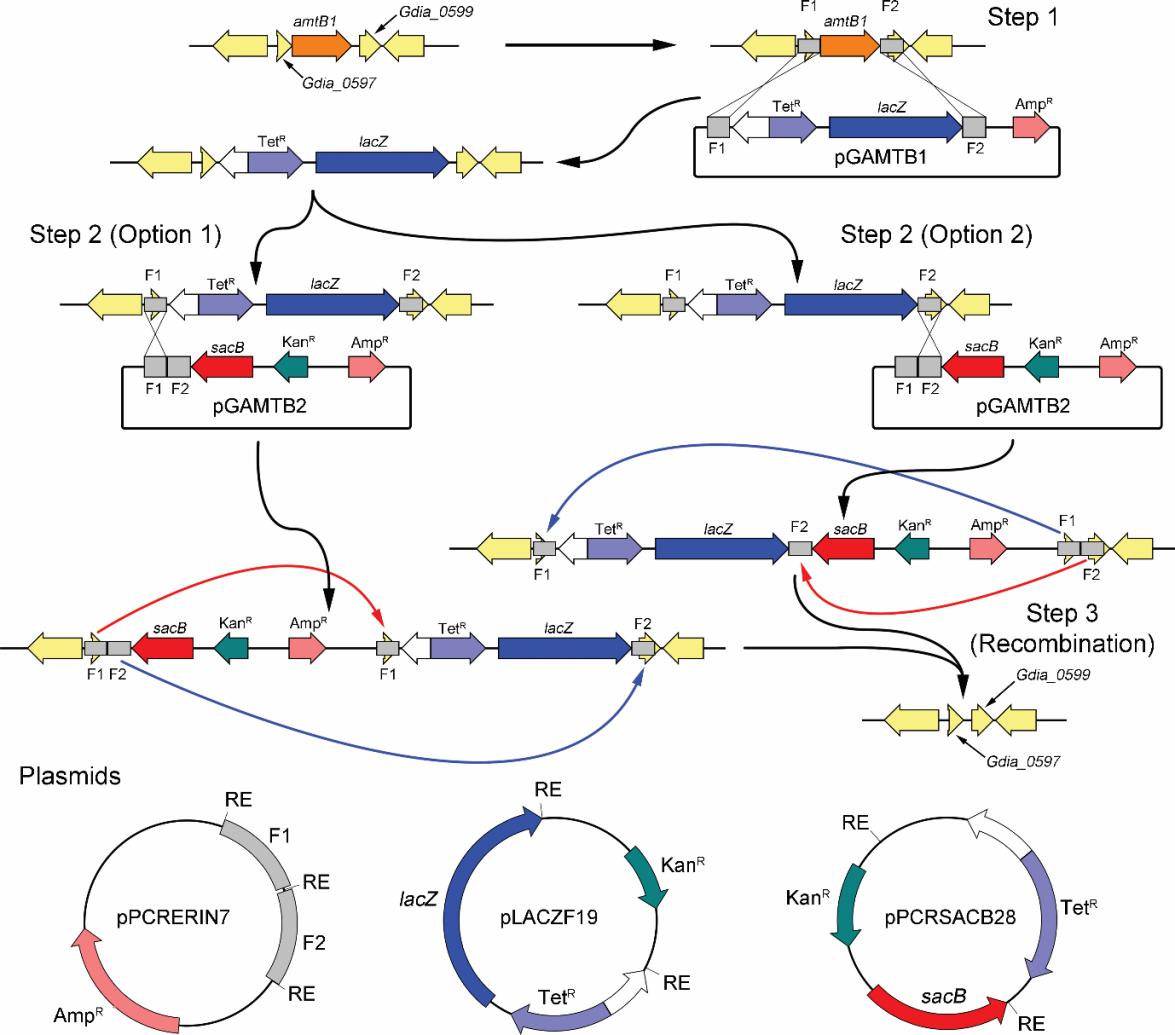
Shown is an illustration of the progression taken to manipulate the genome of *G. diazotrophicus*. In the first step, a section of the genome (*amtB1*) is targeted for gene deletion. Flanking segments F1 and F2 of this gene were cloned into restriction enzyme sites (RE) of pPCRERIN7 (bottom left). In step one, the tetracycline (Tet^R^) selection and *lacZ* (blue color identification) cassette from pLACZF19 were inserted between the flanks of pPCRERIN7 to make pGAMTB1. This plasmid was transformed into *G. diazotrophicus* and double homologous recombinants were selected. Next, pGAMTB2 was constructed by inserting the kanamycin (Kan^R^) selection and *sacB* (conditionally toxic with sucrose) cassette from pPCRSACB28 into the RE adjacent to F2. This plasmid was transformed into the strain with the Tet^R^/*lacZ* cassette and single homologous co-integrants were selected. There are two potential recombination products depending upon if pGAMTB2 has recombined with F1 (Option 1) or F2 (Option 2). Strains produced in step 2 were allowed to grow without antibiotic pressure for two days to allow flanking regions to recombine and then subjected to sucrose toxicity pressure to select strains that had lost the *sacB* gene. Undesired recombination events are shown in red, while desired recombination events are shown in blue. The desired trait can be selected at this point by growth with X-Gal to look for strains that no longer harbor the *lacZ* gene and the resulting blue phenotype.

Plasmids were constructed within *Escherichia coli* JM109 obtained from New England Biolabs (Ipswich, MA) as previously described (13, 29). Strains constructed for this study are listed in Table 1. The plasmids for the construction of *G. diazotrophicus* strains are described in Table 2. Primers required for the construction of the strains are listed in Table 3. Plasmids were transformed into *G. diazotrophicus* through electroporation as described previously (53, 54). DNA transfer was accomplished by growing *G. diazotrophicus* in liquid GADN medium until it had reached an OD_600_ of approximately 1.0, then 1 mL of cells were spun down at 7,000 × *g* and washed two times with 10% glycerol in distilled water and resuspended in 100 µL. These cells were electroporated in 0.1 cm gap electroporation cuvettes using a BioRad gene pulser set at 600 Ω resistance, 25 µF capacitance, 12.5 kV/cm, and a pulse length of ~12 ms. After electroporation, the cells were transferred to 50 mL of fresh GADN medium in a flask and allowed to grow overnight. Following growth, 1 mL of cells were spun down and plated on GADN plates with selected antibiotics. When utilized, X-gal (50 µL of 40 mg/mL 5-bromo-4-chloro-3-indolyl-β-D-galactopyranoside in DMSO) was spotted on and spread across agar plates. An example of a typical strain construct strategy is shown in Fig. 2.

**TABLE 1:** *G. diazotrophicus* strains constructed for this study

Strain^*a*^	Genotype	Vector (technique)	Reference
**Wild-type**	Wild-type *G. diazotrophicus* PA1 5 obtained from Cedric Owens (ATCC 49037)	None	(48, 50)
GABB019	*nifA*::Tet^R^	Wild-type with pPCRGNIF5	This study
**GABB027**	Δ*Q-linker*	GABB019 with pPCRGNIF3	This study
GABB029	*amtB1*::Tet^R^-*lacZ*	Wild-type with pGAMTB1	This study
GABB030	*amtB1*::Tet^R^-*lacZ* with single homologous recombination of pGAMTB2	GABB029 with pGAMTB2	This study
**GABB031**	Δ*amtB1*	Recombination of GABB030	This study
GABB032	*amtB2*::Tet^R^-*lacZ* Δ*amtB1*	pGAMTB3 with GABB031	This study
GABB033	*amtB2*::Tet^R^-*lacZ* with single homologous recombination of pGAMTB4*,* Δ*amtB1*	pGAMTB4 with GABB032	This study
**GABB034**	Δ*amtB1* Δ*amtB2*	Recombination of GABB033	This study
GABB037	*Q-linker*::Tet^R^-*lacZ* Δ*amtB1* Δ*amtB2*	pPCRGNIF12 with GABB034	This study
GABB039	*Q-linker*::Tet^R^-*lacZ* with single homologous recombination of pPCRGNIF14 Δ*amtB1* Δ*amtB2*	pPCRGNIF14 with GABB037	This study
**GABB040**	Δ*Q-linker* Δ*amtB1* Δ*amtB2*	Recombination of GABB039	This study

^*a*^ Strains in bold are completed strains containing no antibiotic markers that were used for the analysis of elevated nitrogen production.

**TABLE 2:** Key plasmids used in this study

Plasmid^*a*^	Relevant gene(s) and approach	Parent vector	Source or reference
pPCRERIN2	Cloned *amtB1* and flanking regions from *G. diazotrophicus* into pBB053	pBB053	(55)
pPCRERIN5	Cloned *amtB2* and flanking regions from *G. diazotrophicus* into pBB053	pBB053	(55)
pPCRERIN7	Plasmid containing two DNA segments flanking regions for *amtB1* in *G. diazotrophicus*	pPCRERIN2	This study
pPCRERIN10	Plasmid containing two DNA segments flanking regions for *amtB2* in *G. diazotrophicus*	pPCRERIN5	This study
pPCRSACB20	Moved kanamycin cassette into plasmid containing *sacB* gene to construct new cassette with both genes	pPCRSACB7 and pPCRKAN4	(55)
pPCRSACB28	Made various modifications to reorganize *sacB* gene behind the kanamycin resistant gene as a single cassette for simple transfer to other constructs	pPCRSACB20	This study
pLACZF19	Moved *lacZ* gene from pLACZF12 downstream of the tetracycline resistant gene in pBBTET6 to create cassette containing tetracycline resistance and *lacZ*	pLACZF12 and pPCRTET6	(13, 51)
pGAMTB1	Plasmid containing the *lacZ* and tetracycline resistance cassette from pLACZF19 inserted between the flanking regions of *amtB1*	pPCRERIN7 and pLACZF19	This study
pGAMTB2	Plasmid containing the *sacB* and kanamycin resistance cassette from pPCRSACB28 inserted outside of the second flank for *amtB1*	pPCRERIN7 and pPCRSACB28	This study
pGAMTB3	Plasmid containing the *lacZ* and tetracycline resistance cassette from pLACZF19 inserted between the flanking regions of *amtB2*	pPCRERIN10 and pLACZF19	This study
pGAMTB4	Plasmid containing the *sacB* and kanamycin resistance cassette from pPCRSACB28 inserted outside of the second flank for *amtB2*	pPCRERIN10 and pPCRSACB28	This study
pPCRGNIF1	Cloned *nifA* Q-linker and flanking regions from *G. diazotrophicus* into pBB053	pBB053	(55)
pPCRGNIF2	Plasmid containing two DNA segments flanking Q-linker of *nifA* in *G. diazotrophicus*	pPCRGNIF1	This study
pPCRGNIF3	Performed PCR to remove Q-linker region from *nifA* in DNA segment from *G. diazotrophicus*	pPCRGNIF1	This study
pPCRGNIF5	Plasmid containing tetracycline selection marker from pBBTET6 inserted between flanking regions of pPCRGNIF2	pPCRGNIF2 and pBBTET6	(51)
pPCRGNIF12	Plasmid containing the *lacZ* and tetracycline resistance cassette from pLACZF19 inserted between the flanking regions of the Q-linker for *nifA*	pPCRGNIF2 and pLACZF19	This study
pPCRGNIF14	Plasmid containing the *sacB* and kanamycin resistance cassette from pPCRSACB28 inserted outside of modified DNA segment containing *nifA*	pPCRGNIF3 and pPCRSACB28	This study

^*a*^ All plasmid sequences are available upon request.

**TABLE 3:** Primers used in this study

Primer	Sequence (5′ to 3′)	Purpose
BBP1747	CACATGTTCT TTCCTGCGTT ATCCC	Confirmation of *lacZ* insertions
BBP2161	CGCCTAGCTT CCTGCTGAAC ATC	Confirmation of *sacB* insertions
BBP3158	NNNGAATTCG TGCTGCCAGC CCATAAATCA GG	Cloning *amtB1* and flanking regions
BBP3159	NNNTCTAGAC GACTCGCTGG AATATTTGGG CGACAC	Cloning *amtB1* and flanking regions
BBP3160	NNNGGATCCG AATTCTGTGC ATATGGCGTC TTCTCCCGTC TCGC	Delete *amtB1* from plasmid
BBP3161	NNNGGATCCC TGATCCATTT CACCGAACAA CAGACAGG	Delete *amtB1* from plasmid
BBP3184	NNNGAATTCC TTTTTTATTT CACAATCGAT GCAACCAGCG TTTCC	Cloning *amtB2* and flanking regions
BBP3185	NNNAAGCTTC TTCGTCAGCC ATGGCAGGTG GGTATC	Cloning *amtB2* and flanking regions
BBP3164	NNNGGATCCG AATTCGTTCA TATGGCAATC TCCCCGGTTT CATTCGTACG GATAC	Delete *amtB2* from plasmid
BBP3165	NNNGGATCCT GATCGTTCCG GAAACGGGC	Delete *amtB2* from plasmid
BBP3188	GATCGTCTCA TGGGCACTCA GTGAC	Confirmation of *amtB1* manipulations
BBP3189	GTGTATCAGA CCGTTCCGCT GCAG	Confirmation of *amtB1* manipulations
BBP3192	GAATGACGAG ACGTACAAGG TCGCCATGAC	Confirmation of *amtB2* manipulations
BBP3193	CGATAGATTT CGCCGCGATA ATTGAAGG	Confirmation of *amtB2* manipulations
BBP3269	NNNTCTAGAC TGGTGAGGTG GAGGAGGCAA GTAG	Cloning Q-linker and flanking regions of *nifA*
BBP3270	GGTCAGCGAC GTCCGGTTGG TTTCG	Cloning Q-linker and flanking regions of *nifA*
BBP3271	CAGGTTTTCC CGGTCCCGCT GCAC	Removal of Q-linker
BBP3272	GCGCCGGTTG CCGATGGCGG	Removal of Q-linker
BBP3273	NNNGGATCCC AGGTTTTCCC GGTCCCGCTG CAC	Deletion of Q-linker region
BBP3274	NNNGGATCCG CGCCGGTTGC CGATGGCGG	Deletion of Q-linker region

### Deletion of *amtB* genes

Using two steps that first replace an *amtB* gene with a visual marker (*lacZ*) through a double homologous recombination event and then introduce the conditionally toxic *sacB* gene through single homologous recombination (56), we constructed a strain containing multiple flanking regions with a strong potential for deleting the visible and selectable markers (Fig. 2). The desired deletion was identified by growing in GADN medium two times without antibiotic pressure and then transferred to medium supplemented with 20% sucrose. High levels of sucrose were required to achieve a *sacB*-dependent growth deficiency in *G. diazotrophicus* (56, 57). After selection, deletions were identified by plating on GADN plates containing X-Gal to identify colonies that no longer carried the *lacZ* gene and linked antibiotic markers and then confirmed by PCR. Applying this approach, we constructed clean gene deletions for both *amtB1* and *amtB2* (Gdia_0598 and Gdia_1303, respectively). These two *amtB* genes were deleted sequentially and were confirmed by PCR with primers external to the manipulated regions and then by antibiotic challenge to confirm loss of the antibiotic markers in the final construct. For the completed constructs, the entire gene was removed from these strains, confirming that the desired modification was successful in each case.

### Modifications to the Q-linker region of *nifA*

In addition to the ability to remove genes in their entirety with no remaining antibiotic markers, the genome editing method described here also enables additional and more strategic modifications or even the introduction of new genes, again without leaving an antibiotic marker behind. This is because manipulations made to the plasmid in the single homologous second recombination step of the genome editing method (step 2 in Fig. 2) are incorporated into the genome following the internal recombination under selective pressure based on sucrose in combination with *sacB*. This was demonstrated by first removing a small segment of the *nifA* gene from *G. diazotrophicus* using the double homologous approach in step one of the gene editing protocol. The plasmid containing the flanking regions was further modified to construct the desired genome modification, which here was the removal of a series of codons encoding the Q-linker region of *nifA* (58). Once confirmed, the *sacB* and kanamycin cassette were again added to the plasmid outside of the segment containing the desired modifications and flanking regions to the manipulated Q-linker region. In this manner, internal recombination during the final step of the gene editing method that removes the *lacZ* gene and tetracycline marker also introduces the newly manipulated region back into the genome in a seamless manner. This was confirmed both by itself (GABB027) and in combination with the dual *amtB* deletion strain (GABB040) by PCR of the final genome segment. Following confirmation by PCR, the segment of the genome containing the Q-linker was sequenced by Sanger sequencing to confirm removal of the specific codons coding for the Q-linker amino acids (DRENL**LHDSGLAQPA**APVAD to DRENLAPVAD).

### Algal and *G. diazotrophicus* co-culture growth

Following confirmation of the various strains constructed as part of this work, *G. diazotrophicus* strains were tested for elevated nitrogen production. Elevated nitrogen production was screened by growing *Chlorella sorokiniana* UTEX 1602 (59) in the presence of *G. diazotrophicus* without any supplemented nitrogen present in the growth medium. A similar method has been previously applied where *A. vinelandii* was grown with *C. sorokiniana* (13).

**Fig 3:**
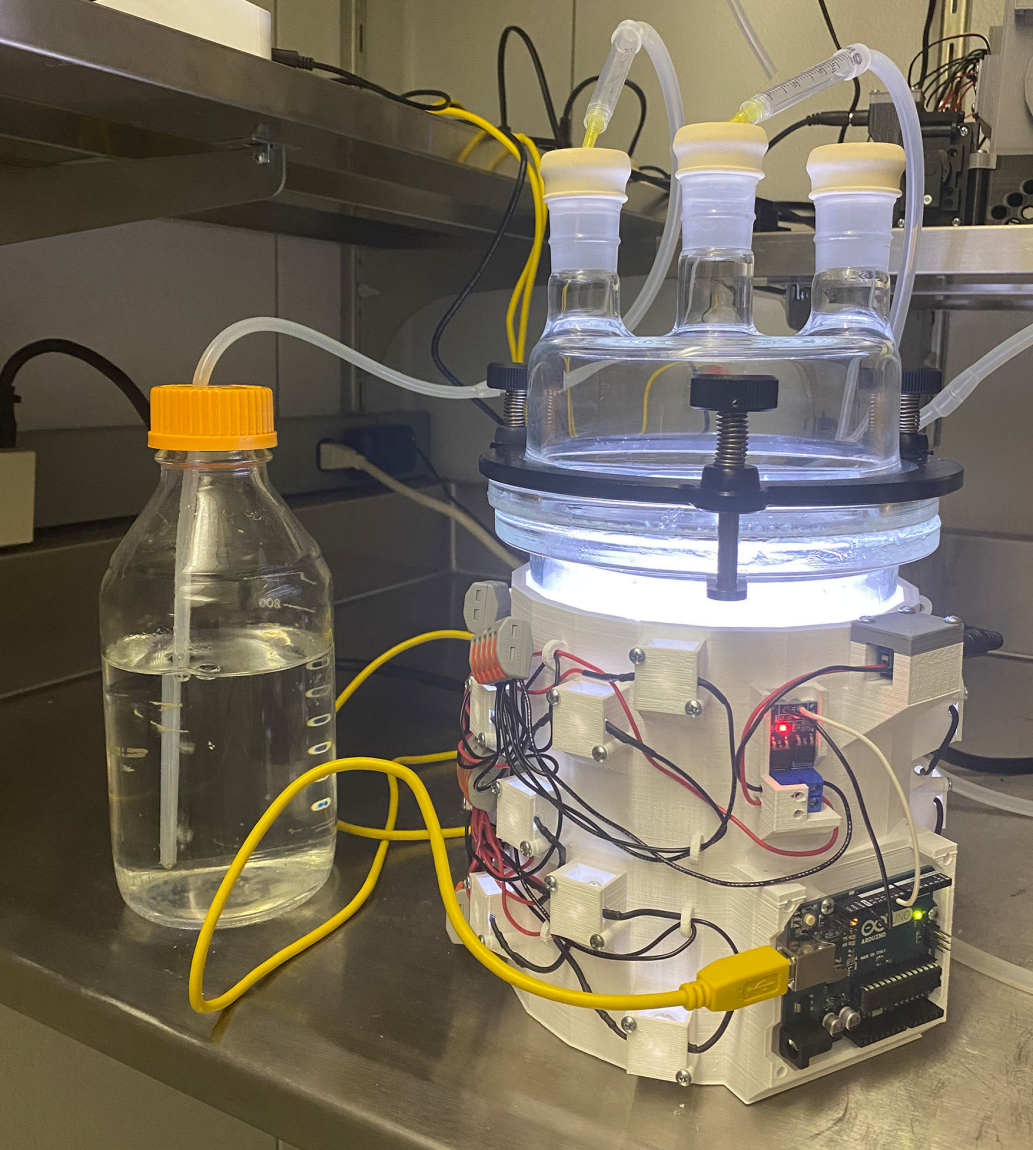
Shown is an image of the micro-aerobic chamber with supplemented LED lights used to co-culture *G. diazotrophicus* strains and *C. sorokiniana*. Lights were turned on and off on an 8-hour cycle.

To culture *G. diazotrophicus* and *C. sorokiniana* together, cells were first grown separately on their respective medium. After three days of growth on GADN plates, 20 µL (1/3 loop) of *G. diazotrophicus* was suspended in 0.5 mL of distilled water. *C. sorokiniana* cells were grown on freshwater plates for one week, and then a similar quantity of cells was scraped and washed twice before resuspending in 0.5 mL of distilled water. Cells of each culture were diluted 20 fold in distilled water and 50 µL was spotted onto solid agar of the modified Burk’s medium and allowed to absorb. The spots were grown in a micro-aerobic chamber at 5% oxygen in a 95% nitrogen background. The growth apparatus is shown in Fig. 3. Gases are maintained at a constant flow through mass flow controllers and exhaust gas is bubbled through a flask to create a positive pressure barrier between the external atmosphere.

### Ammonium quantification of individual strains

To measure the accumulation of ammonium, *G. diazotrophicus* strains were first inoculated from fresh GADN plates into 125 mL Erlenmeyer flasks containing 50 mL of GADN medium and grown to an OD at 600 nm of ~2.0 to 2.5. Then 1 mL of culture was removed and the cells pelleted at 12,000 *g* for 1 minute, and the supernatant was removed. The pellet was resuspended in 1 mL of the modified Burk’s medium listed below and transferred to a new 125 mL Erlenmeyer flask containing 50 mL of the modified Burk’s medium. The modified Burk’s (B) medium contained 20 g sucrose, 200 mg MgSO_4_⋅7H_2_O, 90 mg CaCl_2_⋅2H_2_O, 0.5 mg Na_2_MoO_4_⋅2H_2_O, 10 mg FeSO_4_⋅7H_2_O, 200 mg KH_2_PO_4_, and 800 mg K_2_HPO_4_ in 1 L dH_2_O; supplemented with 35 mM sodium citrate and 1.25 mM asparagine and adjusted to pH 6.8 with HCl or NaOH. The asparagine concentration was selected to allow the culture to reach an OD_600_ of approximately 0.5, at which point the culture was able to grow diazotrophically under fully aerobic conditions at 28°C and 180 rpm and remain in a planktonic state of growth. Flasks were sampled daily for optical density and ammonium quantification. Ammonium was quantified using the o-phthalaldehyde assay protocol as previously described (13, 60). For high ammonium concentrations (>100 µM), a spectrophotometric approach was employed for quantification using a Cary 50 spectrophotometer, while for low ammonium concentrations (<100 µM), ammonium was quantified by fluorescence using a FluoroMax+ fluorometer (Horiba Scientific).

## RESULTS

### Micro-aerobic diazotrophic growth of *G. diazotrophicus*

In order to test for elevated nitrogen production under diazotrophic conditions, *G. diazotrophicus* was grown micro-aerobically. *G. diazotrophicus* requires micro-aerobic conditions to grow diazotrophically on a solid medium as the nitrogenase enzyme is sensitive to oxygen (61). In previous studies, we grew liquid cultures of *G. diazotrophicus* at 2.5% O_2_ (remainder N_2_) to enable diazotrophic growth (51). However, when grown on solid agar plates, we found that diazotrophic growth required a stream of 5% O_2_ in the headspace. We also tested different algal strains to confirm their ability to grow under this low-oxygen environment. *C. sorokiniana* grew well alone under this low oxygen concentration when provided with alternating light and dark cycles (8 hours each) on plates provided with supplemented nitrogen in the form of nitrate.

### External nitrogen production

**Fig 4:**
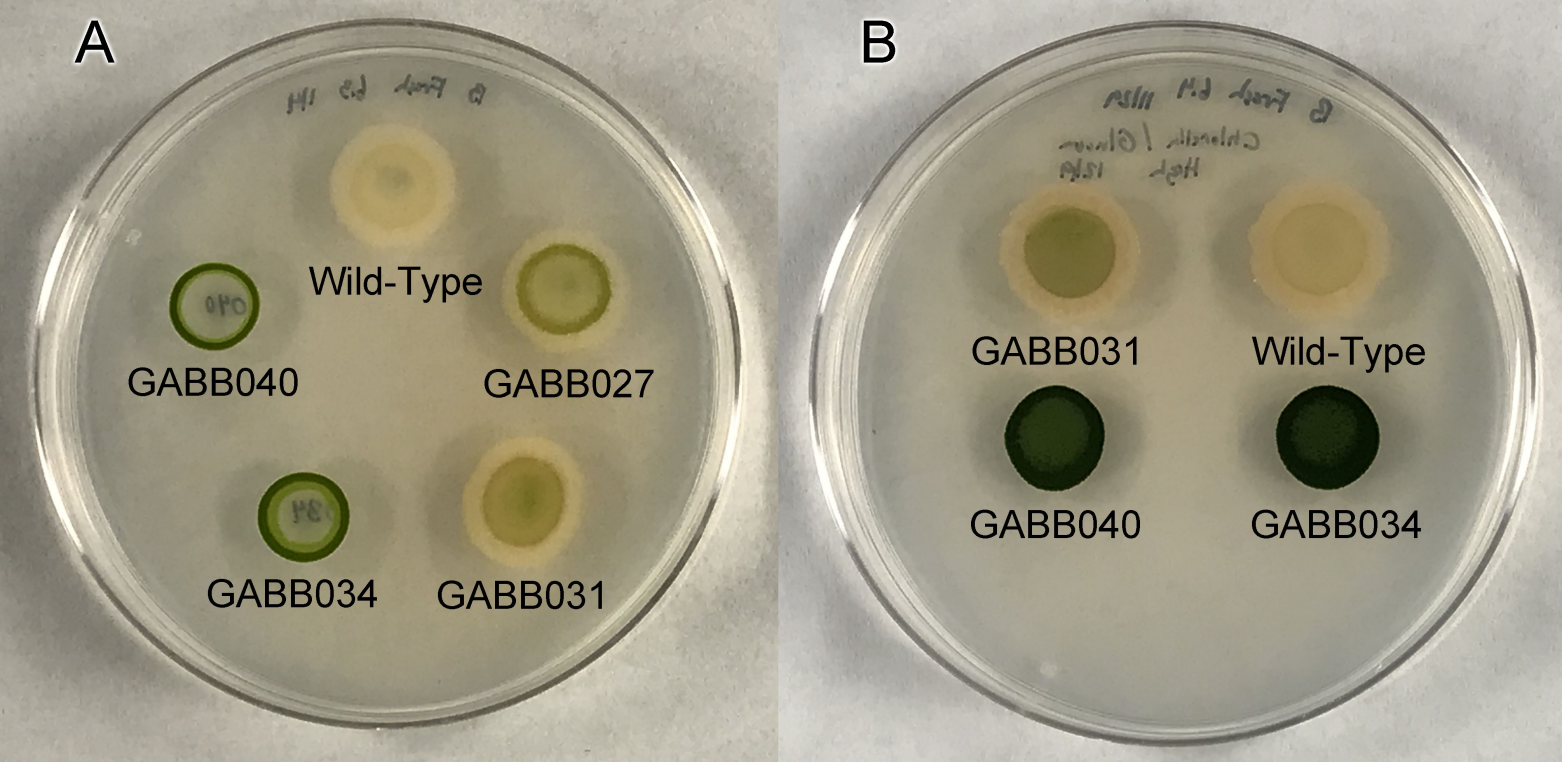
Shown are images of plates of *C. sorokiniana* grown together with wild-type and manipulated strains of *G. diazotrophicus*. Image A shows selected strains following 10 days of growth. Image B shows selected strains following three weeks of growth. GABB027 is the ΔQ-linker, GABB031 is Δ*amtB1*, GABB034 is Δ*amtB1* Δ*amtB2,* and GABB040 is ΔQ-linker Δ*amtB1* Δ*amtB2*.

Since *C. sorokiniana* requires nitrogen to grow and produces green photosynthetic pigments, it can be used as a biosensor for available nitrogen, and can be distinguished from *G. diazotrophicus* in combination on plates (Fig. 4). The wild-type strain generates minimal extracellular nitrogen, resulting in a slow bleaching of the algal cells over the course of a week. The single *amtB1* deletion (GABB031) and the Q-linker deletion (GABB027) result in minimal growth of *C. sorokiniana*, indicating slight improvement in external nitrogen release. The dual *amtB* deletion strain (GABB034) and the combined Q-linker with the dual *amtB* deletion (GABB040) showed a significant difference in the phenotype with *C. sorokiniana*. This change in phenotype indicates an increase in external nitrogen since the growth medium in the plates is devoid of fixed nitrogen. In addition to an increase in extracellular nitrogen, these strains also produce a lower amount of extracellular polysaccharides, manifesting as a less goopy phenotype that protrudes outside of the initial spotted cells (Fig. 4).

### Extracellular ammonium quantification

Initial growth of *G. diazotrophicus* under diazotrophic conditions requires a micro-aerobic atmosphere to maintain the culture in planktonic form. As described previously, *G. diazotrophicus* has been cultured in a turbidostat reactor that allowed us to maintain a micro-aerobic atmosphere over the culture during the initial stage of growth (51). Under these conditions with a low oxygen atmosphere, cultures achieved a cell density of approximately 0.1 to 0.2 OD_600_. Following further investigation in this work, we determined that diazotrophic growth to higher densities than 0.2 OD_600_ was not possible under these conditions unless the percent oxygen concentration of the atmosphere was incrementally increased. Once the atmosphere was shifted to a standard atmosphere with a slow flow rate, cell densities of approximately 0.4 to 0.5 OD_600_ were possible in the turbidostat, with the majority of the culture remaining in a planktonic phase of growth. These findings differ from prior reports that have characterized nitrogen fixation in *G. diazotrophicus* (33, 61, 62) and indicate that nitrogen fixation (and as a result, culture density) are oxygen-limited at higher density, but require micro-aerobic conditions at low density to transition from non-diazotrophic growth conditions. In addition to requiring elevated densities, the cells also grew better if they remained in a planktonic phase of growth, as they were prone to form aggregates if growth was slow through this phase. Reversing the culture back to a planktonic growth phase was difficult under diazotrophic growth once the culture had begun to aggregate.

**Fig 5:**
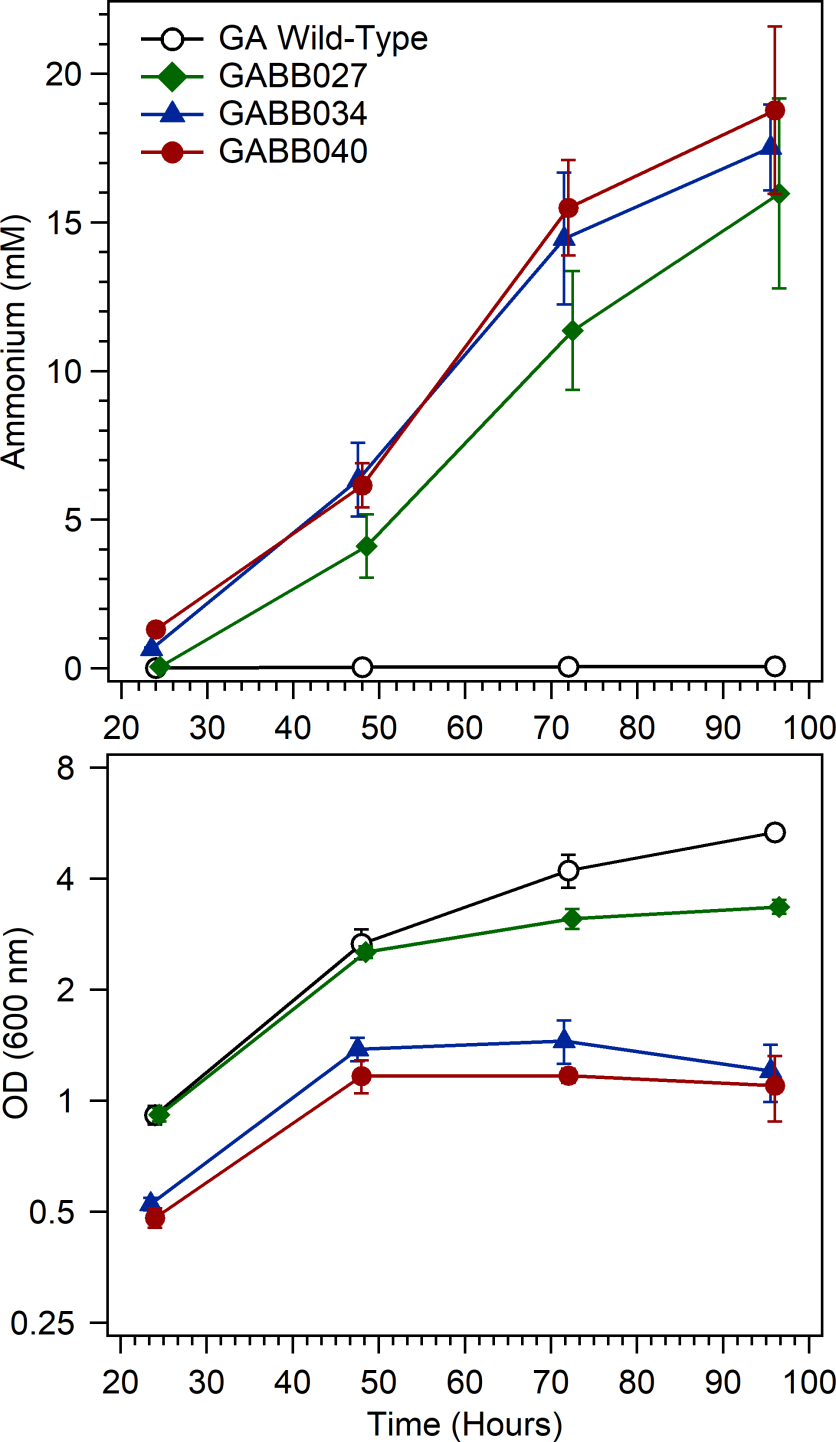
Ammonium accumulation over time during aerobic diazotrophic growth of various *G. diazotrophicus* strains. *G. diazotrophicus* strains were grown in Erlenmeyer flasks following the transfer of planktonic cells to a medium containing limited asparagine (1.25 mM). The upper plot shows ammonium levels obtained at different time points. The lower plot shows the corresponding OD_600_ plotted on a Log2 scale. Cultures were grown at 180 rpm and 28°C under a standard atmosphere. Results represent the average and standard deviation for at least three samples. Sampling times were offset by 0.5 hour in certain cases to better illustrate the standard deviation for individual strains. Starting ODs were approximately 0.04 at 600 nm. Cultures remained in planktonic growth through 72 hours, but GABB034 and GABB040 began to show signs of aggregation at 96 hours. GABB027 is the ΔQ-linker, GABB034 is Δ*amtB1* Δ*amtB2* and GABB040 is ΔQ-linker Δ*amtB1* Δ*amtB2*.

To overcome the issues associated with transitioning cultures from micro-aerobic conditions in turbidostats to fully aerobic growth and also maintain the cells in a planktonic state of growth, we developed a protocol to transition the cells to an OD_600_ of approximately 0.5 by transferring a small aliquot of cells from GADN medium (in planktonic growth) to a medium containing a limiting amount (1.25 mM) of asparagine, which can serve as a minimal nitrogen source. Once cells achieved this 0.5 OD_600_ (in the absence of any aggregates), the cells were able to grow diazotrophically under aerobic conditions, in a manner similar to the conditions used to generate extracellular ammonium from *A. vinelandii* (26). Strains GABB027 (ΔQ-linker), GABB034 (Δ*amtB1*, Δ*amtB2*), and GABB040 (ΔQ-linker, Δ*amtB1*, Δ*amtB2*) all achieved high levels of ammonium (16–19 mM) after four days of growth (Fig. 5), while wild-type *G. diazotrophicus* remained below 0.1 mM ammonium throughout the entire 4 days. These quantities well exceed the potential nitrogen that could be derived from the 1.25 mM of asparagine used to support the initial growth of the cells while transitioning to aerobic diazotrophic growth. Cell density for wild-type *G. diazotrophicus* achieved an OD_600_ of 5.3 after four days, while GABB027 achieved 3.3, and GABB034 and GABB040, respectively, reached only 1.4 and 1.1 OD_600_. These results correlate well with the algal co-culture plate experiments and revealed that levels of ammonium achieved for GABB027, GABB034, and GABB040 were considerably higher than what could be achieved with wild-type, with minor growth defects found for increasing complexity of the gene deletions, manifesting as a lower final cell density achieved.

## DISCUSSION

The purpose of this work was to develop an elevated nitrogen-producing endophyte diazotrophic strain for potential future application as a biofertilizer and compare nitrogen levels obtained to other modified microbes. Prior studies in *A. vinelandii* have demonstrated multiple approaches to increase extracellular nitrogen production in a free-living diazotroph (13, 23, 26, 27, 44). The endophyte *G. diazotrophicus* was engineered to produce elevated levels of extracellular nitrogen as ammonium. Genetic modification may raise concern due to the potential to introduce foreign genes into the environment, often in the form of antibiotic resistance used for selection during strain construction. Although the strains constructed here were genetically altered, we used an approach that generates clean gene deletions (Fig. 2). Clean deletions do not leave any antibiotic-resistant markers behind in the final strain. The strain simply has genes removed that were originally obtained through horizontal gene transfer or evolution. The removal of genes often results in a disadvantage for the bacteria, as was shown previously for *amtB* disruptions in Tn-seq data, giving the wild-type strain an advantage in the environment (51). The wild-type strain should out-compete this modified strain, resulting in an eventual decline in numbers for manipulated strains. Hence, there should be minimal concern for this strain if applied as a biofertilizer. Because an entire gene is removed, the potential to reacquire the entire gene represents a significant barrier.

The selection of *C. sorokiniana* as a surrogate for higher land plant studies was done here to capitalize on the rapid growth rate and ability to test the strain on solid medium under micro-aerobic conditions within a sealed chamber. *C. sorokiniana* is unable to fix nitrogen, and is thus dependent on *G. diazotrophicus* for nitrogen, serving as a biosensor for extracellular nitrogen. Amounts of ammonium accumulated in the medium as a result of ammonium transport abolishment have been reported to be very low, in the μM range in liquid culture for other strains (13, 18, 43), so the use of a biosensor is a convenient alternative for measuring low levels of ammonium release by growing the two strains in close proximity (13). Elevated levels of algal growth for the Q-linker deletion strain GABB027 indicate that disruption of the NifA Q-linker alone in *G. diazotrophicus* is sufficient for a minimal increase in extracellular ammonium under micro-aerobic conditions, as has been demonstrated previously in *R. palustris* and *R. sphaeroides* (45, 47, 58).

When grown to high density under aerobic growth, the disruption of the NifA Q-linker (Fig. 1) was sufficient to yield high levels of ammonium, similar to what was obtained with more extensively manipulated constructs such as GABB040. Actual concentrations of ammonium that accumulated in *G. diazotrophicus* for the dual *amtB* deletion strain GABB034 achieved levels of ammonium surpassing 17 mM under optimal conditions, even without the modifications to the Q-linker of *nifA*. This is significantly higher than what has been reported for *amtB* disruptions in other strains (13, 18, 43), where levels of ammonium only achieved low μM concentrations. The combination strain of the dual *amtB* gene deletion with the Q-linker disruption achieved a similar ammonium concentration of 18 mM. Reasons why the *amtB* dual deletions alone had such a large impact on extracellular ammonium release versus prior strains could relate to the growth conditions and culture pH, or to differences in cell wall composition making this strain more susceptible to ammonium or ammonia diffusion. This might partially explain why this microbe has two *amtB* homologs within its genome to counter the higher susceptibility to ammonium loss. This loss of intracellular ammonium combined with the missing AmtB partners for the PII regulatory proteins (41) may result in increased nitrogenase activity that fuels further fixed nitrogen losses to the environment. This may also be a general feature of strains lacking a NifL component of the NifLA regulatory system. Other diazotrophs also contain multiple *amtB* homologs. These potential explanations for these stark differences are all speculative and would require additional testing in other diazotrophic microbes lacking a *nifL* gene.

The *nifA* Q-linker disruption alone (GABB027) resulted in similar, but slightly lower ammonium yields, though the differences between GABB027, GABB034, and GABB040 are minimal in terms of ammonium production (Fig. 5). These levels are approaching the levels of 25–30 mM ammonium that has been achieved for *A. vinelandii* in previous reports (23, 24, 26, 63). Our experiments demonstrate that *G. diazotrophicus* requires either a phase of growth under micro-aerobic conditions to achieve sufficient cell density in a planktonic form or a minimal quantity of a nitrogen source during the transition to diazotrophic growth to prime the culture for aerobic diazotrophic growth. Once a sufficient density of planktonic cells is achieved, *G. diazotrophicus* can be grown to higher density under a standard aerobic atmosphere (Fig. 5), indicating that the cells actually require elevated oxygen to grow to high density as a diazotroph, similar to what is required for *A. vinelandii* (26). For this reason, we believe it is more appropriate that *G. diazotrophicus* be characterized as conditionally micro-aerobic in contrast to micro-aerobic for diazotrophic growth, as it is generally classified. The development of this approach for testing the diazotrophic growth of *G. diazotrophicus* without the need for a micro-aerobic atmosphere should be useful for future assays of this microbe, as it is highly reproducible and easy to scale without a need for atmospheric control (Fig. 5).

Testing the potential of these modified biofertilizer strains with higher land plants is an obvious next step. Successful application of this strain as a fertilizer may require annual inoculation due to the slightly diminished growth rate versus wild-type strains. This decreased rate of growth is less noticeable when grown on a rich medium such as GADN, where strain growth is indistinguishable from wild-type. Potential methods to apply the strain might vary and will require further study as well. It is thought that *G. diazotrophicus* enters the plant through cracks at lateral root emergence sites (37). This means that *G. diazotrophicus* could be applied to the seeds just prior to planting, allowing them to enter the plant during the early stages of growth and live in the plant throughout its lifespan. *G. diazotrophicus* could also be applied to the soil during early plant growth, allowing it to enter the plant at this time. Another way endophytes can be introduced to plants is through the stomata in the leaves of the plant (37). This would allow *G. diazotrophicus* to be applied to the plant at later stages, potentially by application using precision farm equipment. Our results indicate significant improvements in extracellular ammonium production for *G. diazotrophicus* that can be monitored using either a microalga as a biosensor on solid plates under micro-aerobic conditions or by direct methods to quantify ammonium, even when grown under a standard atmosphere.

The characteristics of *G. diazotrophicus* as a growth-promoting endophyte (33, 48, 50) and the application of genetic approaches here and elsewhere (64) for its manipulation provide multiple opportunities to further tailor this strain to improve specific functions. In this work, we demonstrate that the deletion of the two ammonium-transporting genes along with removal of the Q-linker from NifA increases the amount of nitrogen released outside of the cell. It also demonstrates that the amount of ammonium that can be obtained from *G. diazotrophicus* using only the dual *amtB* deletions is far higher than what has been reported for other strains (13, 18, 43, 65). We also demonstrated a method for clean deletions using *lacZ* as part of the counter-selection protocol to help identify desired strains in *G. diazotrophicus*. This method could apply to a broad range of additional bacteria as well. Future experiments with specific higher land plants will determine the full potential of these different strains, while results presented here demonstrate that these strains of *G. diazotrophicus* have increased extracellular release of nitrogen as ammonium, reaching levels that are similar to what has been achieved with *A. vinelandii* manipulated strains (21, 26, 44).
